# Cervical Chondrocutaneous Branchial Remnants: A Case Report

**Published:** 2016-05-09

**Authors:** Lea Juul Nielsen, Kasper Von Rosen, Linda Plovmand Jakobsen

**Affiliations:** Department of Plastic Surgery, Breast Surgery and Burns Treatment, Rigshospitalet, Copenhagen, Denmark

**Keywords:** cervical, chondrocutaneous, branchial, remnants, choristoma

**Figure:**
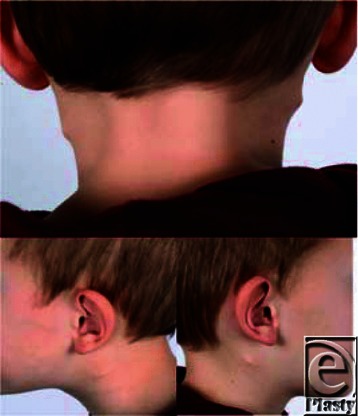


## DESCRIPTION

A 5-year-old healthy boy presented with cervical tumors, present since birth, causing only cosmetic complaints. Physical examination showed bilateral tumors, free from underlying structures, and no other deformities. Abdominal and cardiac ultrasound studies showed no anomalies. A brother presented with a similar, unilateral tumor. Histology after excision showed hyaline cartilage.

## QUESTIONS

1. **Which diagnosis should be suspected when looking at the pictures and what are the objective and histological findings?**
2. **What is the embryological development connected to this condition?**
3. **Are further investigations indicated?**
4. **Which treatment should be offered?**

## DISCUSSION

Cervical chondrocutaneous branchial remnants (CCBRs) are rare, benign neck tumors, first reported in 1858.^1^ To date, 104 cases have been reported, 28 with bilateral lesions.^2^ We have found a further 13 cases (6 bilateral) totaling 117 cases. The majority of unilateral cases are left-sided. Until now, a marked predominance of males has been reported, but in a recent review, no gender predisposition was found,^2^ which was confirmed in the present literature search. The lesions are present at birth and exhibit no or very slow growth. They are typically located in the middle or lower third of the neck, anterior to or over the sternocleidomastoid muscle. The lesions consist of normal skin with a cartilage core. No connection to underlying deep structures has been reported, but adherence to the fascia of the sternocleidomastoid muscle is often reported. There have been no reports of underlying sinuses or cysts. Histologically, CCBRs are defined as a choristoma, which is a mass of tissue that is histologically normal for an organ or a tissue but foreign to the tissue or site at which it is located.^3^ The pathogenesis of CCBRs is controversial. The presence of elastic cartilage may suggest an auricular origin (from the first or second branchial arch), whereas the presence of hyaline cartilage excludes an auricular origin and suggest a cervical origin (from the second or lower branchial arches). Elastic cartilage is reported in 28 cases and hyaline cartilage in 12 cases. Most of the literature on the subject agrees that the origin must be the second branchial arch. One proposed, and widely accepted, theory is that CCBRs are the result of incomplete obliteration of the branchial apparatus leaving cells behind in the neck during embryonic migration that differentiate into cartilage.^4^ Others suggest that it is rather the presence of pluripotent cell rests, much like the presence of supernumerary nipples, which proliferate into cartilage.^5^

In the fourth week of embryogenesis, the neural crest cells migrate into the future head and neck forming 6 branchial arches. In the fifth week, the auricle develops around the first and second arch and 6 auricular hillocks appear in the sixth week. During the seventh week, the auricular hillocks start to enlarge and differentiate. The auricle starts to translocate from its initial ventral position on the lower side of the lateral neck to its ultimate lateral cranial destination. During this migration, it follows the anterior border of the sternocleidomastoid muscle.^6^

CCBRs have proven to be visible markers for more serious anomalies. Approximately one-third of patients with CCBRs have associated anomalies,^2^ although the prevalence varies and has been found to be as high as 76.^4^ Anomalies reported include auditory, gastrointestinal, genitourinary, cardiovascular, musculoskeletal, visual as well as complex syndromes.^1^^,^^4^^,^^7^ CCBRs can be familial as reported by Pham Dang et al^8^ and in the present case but are predominantly sporadic. It is recommended to initiate investigations with a thorough physical examination, full anamnesis including family history and ultrasound study of the abdomen and heart.

Treatment is simple surgical excision. It is not recommended to shave the lesion but to remove it completely. Treatment is recommended before school age for social reasons and for histopathological verification^1^ but can be postponed to a suitable and safe age.^2^ Neither recurrence nor malignant transformation has been reported.

## SUMMARY

CCBRs are rare, benign malformations usually found in the lower neck, consisting of normal skin with a cartilage core. Since CCBRs can be seen in some rare syndromes and associated anomalies have been reported in up to 76%, a thorough physical examination and ultrasound study of the abdomen and heart are recommended. Treatment is simple surgical excision.
